# Effect of Kerogen Thermal Maturity on Methane Adsorption Capacity: A Molecular Modeling Approach

**DOI:** 10.3390/molecules25163764

**Published:** 2020-08-18

**Authors:** Saad Alafnan, Theis Solling, Mohamed Mahmoud

**Affiliations:** KFUMP King Fahad University of Petroleum and Minerals, Dhahran 31261, Saudi Arabia; mmahmoud@kfupm.edu.sa

**Keywords:** kerogen, thermal maturity, adsorption capacity, source rocks

## Abstract

The presence of kerogen in source rocks gives rise to a plethora of potential gas storage mechanisms. Proper estimation of the gas reserve requires knowledge of the quantities of free and adsorbed gas in rock pores and kerogen. Traditional methods of reserve estimation such as the volumetric and material balance approaches are insufficient because they do not consider both the free and adsorbed gas compartments present in kerogens. Modified versions of these equations are based on adding terms to account for hydrocarbons stored in kerogen. None of the existing models considered the effect of kerogen maturing on methane gas adsorption. In this work, a molecular modeling was employed to explore how thermal maturity impacts gas adsorption in kerogen. Four different macromolecules of kerogen were included to mimic kerogens of different maturity levels; these were folded to more closely resemble the nanoporous kerogen structures of source rocks. These structures form the basis of the modeling necessary to assess the adsorption capacity as a function of the structure. The number of double bonds plus the number and type of heteroatoms (O, S, and N) were found to influence the final configuration of the kerogen structures, and hence their capacity to host methane molecules. The degree of aromaticity increased with the maturity level within the same kerogen type. The fraction of aromaticity gives rise to the polarity. We present an empirical mathematical relationship that makes possible the estimation of the adsorption capacity of kerogen based on the degree of polarity. Variations in kerogen adsorption capacity have significant implications on the reservoir scale. The general trend obtained from the molecular modeling was found to be consistent with experimental measurements done on actual kerogen samples. Shale samples with different kerogen content and with different maturity showed that shales with immature kerogen have small methane adsorption capacity compared to shales with mature kerogen. In this study, it is shown for the first time that the key factor to control natural gas adsorption is the kerogen maturity not the kerogen content.

## 1. Introduction

Source rocks have become an important resource for satisfying worldwide energy demands. They are sedimentary formations produced through massive stimulation operations. A combination of innovative drilling and completion technologies has unlocked the tremendous potential of hydrocarbon resources that for a long time were believed to be inaccessible. However, there is still a gap between our ability to produce those resources and our actual understanding of their petrophysical properties.

Source rock lithologies include but are not limited to shale, mudstone, and coalbed methane. What characterizes them is their mineralogical composition, depositional environment, and, very importantly, the type and amount of kerogen. The word “kerogen” is a common descriptor for complex networks of aromatic and aliphatic hydrocarbon structures with the odd occurrence of heteroatoms substituted into the hydrocarbon framework. Such hydrophobic macrostructures have the capacity to store hydrocarbons both adsorbed on graphite-like carbon frameworks and free compartments; the relative importance of these two storage mediums varies depending on the structure and amount of the kerogen. In a storage capacity context, natural and hydraulic fractures are relatively large. They serve as means of transporting and storing compressed gas. In kerogen, the voids are made up of organic nanopores with the capacity to store significant quantities of gas in the sorbed form because their nanometer size is associated with a significant surface area [1,2,3,4,5,6,7,8,9]. As source rocks are exposed to various types of stimulation procedures, the natural gas is subject to continuous expansion in the larger pores. Desorption in the smaller pores becomes augmented by the induced pressure gradient. During their transition from the rock matrix to the wellbore, natural gas molecules are subjected to transport and storage mechanisms that deviate from those applicable to classical reservoirs. These intricacies have implications for the methods used for reserve assessments. Conventional volumetric and material balance equations MBE have been found insufficient for organic-rich rocks, and much effort has been put into updating them to accommodate the multi-physical nature of natural gas in nanoporous networks [2,10,11,12,13,14,15,16,17,18].

Ambrose et al. [2] derived a volumetric equation that can be used to estimate the initial gas in place. Their approach was based on quantifying sorbed gas with a Langmuir isotherm and free gas with a typical volumetric equation, with the porosity corrected for the volume that constitutes the adsorbed gas. King [10] derived a material balance equation with a form similar to the conventional equation, with some additional terms employed to describe the adsorption. Other researchers have presented similar equations with various degrees of complexity [11,12,13,14,15,16,17,18]. In general, reserve estimation methods for source rocks were conceptualized on dividing reservoir units into inorganic and organic constituents. The former is modelled similar to the classical hydrocarbons traps while the latter is described with some adsorption parameters that are approximated empirically. Reserve estimation methods have various forms based on their underlying assumptions such as the extent of adsorption behavior, the presence of water derive, and the specific type of source rocks. Some equations that are listed in Table 1 have wider range of applicability over the others, e.g., Clarkson and McGovern [12] have terms that model free and adsorbed hydrocarbons, which makes it applicable for shale and coalbed methane reservoirs. On the other hand, Jensen and Smith [11] considered adsorption as the dominant mechanism that limits its applicability to only coalbed methane reservoir. Therefore, understanding the adsorption behavior is of vital importance to properly select and use a given reserve estimation method.

## 2. Knowledge Gap and Objectives

The Equations listed in Table 1 were written in different formulations, with some variations in the assumptions; reserve estimation was their common objective. They all include terms describing adsorption. Parameters such as the Langmuir volume (*V_L_*), maximum adsorption capacity (*G_SL_*), and molar adsorption capacity (*C_ME_*) are used to model source rock’s capacity to store hydrocarbons in the adsorbed form. Estimation of those parameters is accompanied by a high degree of uncertainty. Factors such as total organic carbon (TOC), fluid type, and the thermal maturity of the kerogen dictate the extent of the adsorption. Total organic carbon (TOC) is used as a measure of organic materials (i.e., kerogens) content. The presence of micropores, which are defined as pores of sizes < 2 nm, have been found to be positively correlated with TOC of source rocks [19]. Micropores increase the porosity and hence the adsorption capacity of reservoir rocks. The concentration of micropores within the organic materials has been experimentally linked with thermal maturity [20]. Zhang et al. [21] found the porosity of shales to be higher in the presence of overmature organic materials (i.e., samples that had a vitrinite reflectance >3.0% R_O_). They attributed the increase in porosity to the high level of maturity. On the contrary, low thermal maturity shales were found to lack significant accumulation of micropores [22]. In summary, experimental investigations showed that the presence of micropores is linked to TOC, with their concentration being determined by the degree of organic materials maturation. However, direct measurements of kerogen’s storage capacity are scarce. The organic constituents (i.e., kerogens) are difficult to isolate by chemical and physical means. Hence, a detailed experimental study of their petrophysical properties is challenging. Conversely, molecular modeling can provide insights into the kerogen’s ability to store hydrocarbons without the need for a physical sample.

A computational approach also has the advantage that the structure can be tailored to answer molecular-level questions. In this work, we follow a molecular modeling approach to investigate the impact of kerogen’s thermal maturity on its adsorption capacity. This approach is explained in the next section, beginning with representing kerogens on a molecular level. We then show how single kerogen molecules can be used to form larger nanoporous structures used to assess the methane adsorption capacity.

## 3. Molecular Approach

The classification of kerogens began in the 1940s. It was observed that the kerogens in coal formations were almost indistinguishable from the matrix itself, whereas those present in other sedimentary rocks were more aliphatic in nature [23,24]. Modern classifications of kerogens are based on an elemental analysis. In particular, the ratios of H/C and O/C indicate the origin, degree of aromaticity and oxidation. Classification is related to the origin of the organic material. For example, type I is of algal origin with the principal propensity to form oil. Kerogens embedded in source rock can be more or less ready to form oil (i.e., more or less mature). There is no direct relationship between maturity level and kerogen type. It depends on factors that are not only related to the molecular structure of the kerogen; factors such as the mineralogy of the source rock and underlying pressure and temperature conditions are also involved. Hence, kerogens can be ordered into different types with some range of maturity level within each type [25,26]. In general, kerogens are classified as types I, II, III, and IV in ascending order of aromaticity [27,28]. Types III and VI are sometimes combined into one class [29]. Type I kerogen is primarily aliphatic (i.e., saturated), with a hydrogen-to-carbon ratio greater than 1.5 and oxygen-to-carbon ratio below 0.1 [30]. Type II, in contrast, contains a larger fraction of cyclic aliphatic and aromatic units, which is reflected on the decreasing ratio of H/C [31]. Type III, which arises from a shallow marine environment, is vulnerable to further oxidation and hence has an increased oxygen content, appearing either as a functional group or part of a cyclic moiety [28,30].

The modeling of kerogens’ molecular structures was first accomplished by Forsman [32] through functional group characterization. He recognized two distinct groups of kerogens, the first comprised of aliphatic chains and the second having condensed aromatic blocks fused by functional groups (either ether or alkoxy). Some hypothetical macromolecular models matching the elemental ratios were approximated based on arbitrary building blocks [33,34,35]. However, the models failed to match the measured density of the kerogen, indicating a lack of representative 3D spatial configurations [36]. Advancements in representing kerogen structures at different maturity levels have been achieved, beginning with the model presented by Vandenbroucke [37] for type I kerogen. That was followed by three structures of type II kerogen at diagenesis, the beginning of catagenesis, and end of catagenesis by Behar and Vandenbroucke [38]. With improvements in computer-assisted techniques, more representative porotypes of kerogen structures have been generated, such as the six different models produced by Ungerer et al. [39]. In general, the aforementioned studies were conducted to generate synthetic models that could reproduce some of the macroscopic properties of kerogen. These studies should not be regarded as exclusive representations of naturally occurring kerogen.

**Figure 1:**
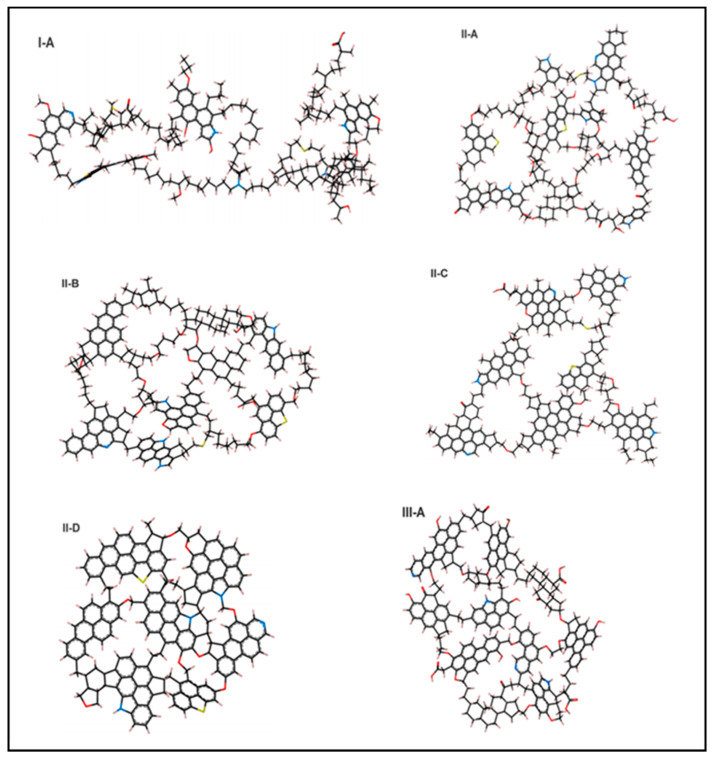
Six prototype kerogen macromolecules [39].

The six prototypes of kerogen (see Figure 1) represent three types. Kerogen type I-A represents an immature macromolecule with mostly plain aliphatic bonding and minimal contributions from cyclic aliphatic and aromatic units. Types II-A, II-B, II-C, and II-D are at a range of maturity levels with increasing degrees of non-aliphatic bonding and polarity to act as anchor points. Type III-A is immature kerogen with a substantial number of aromatic units and high oxygen content. These four type II molecule representations are excellent models for exploring the relationship between kerogen maturity and adsorption capacity. Ungerer et al. [39] proposed that the structures are constructed in such a way that each has a direct relationship to ease, by which they break carbon-carbon bonds and thereby decompose to form hydrocarbons. The macroscopic physical properties of these structures were found to be in a reasonable agreement with their experimental analyses. The principal steering tool is the driving force induced by insertion of heteroatoms to introduce polarity and the introduction of unsaturated units. Variations in the carbon, hydrogen, and oxygen ratios along the bonding types control several chemical and physical properties. In this research, the methane adsorption capacity in particular was investigated. The objective was to further explore the relationship between kerogen’s thermal maturity and its adsorption capacity. The four structures of type II kerogen were utilized in the present research.

Kerogen II-A is immature organic matter with 252 carbon atoms, and its chemical composition is C_252_H_294_O_24_N_6_S_3_. It has two polycyclic saturated structures. The oxygen atoms are associated with ether, carbonyl, hydroxyl, and carboxylic groups in ratios of 9:7:4:2 respectively. Three of the nitrogen atoms are present in thiophenic rings and one in a pyridinic ring. The sulfur is present in two thiophenic rings and one sulfide bridge. The fraction of carbon atoms in aromatic sub-structures is 41%.

Kerogen II-B has a composition of C_234_H_263_O_14_N_5_S_2_ with 45% of its carbon atoms being part of an aromatic system. More oxygen atoms form single bonds with carbon than double bonds compared to type II-A. Nitrogen and sulfur are associated with thiophenic and pyridinic rings.

Kerogen II-C is taken to represent a more mature unit. Its composition is C_242_H_219_O_13_N_5_S_2_. It is very similar to type II-B, but with less alkyl chains to achieve higher maturity level. That is reflected by the percentage of aromatic carbon, which is 58%. Similarly, kerogen II-D is the most mature kerogen type with a composition of C_175_H_102_O_9_N_4_S_2_, and 79% as the percentage of aromatic carbon.

## 4. Building the Kerogen Model Using MD Simulation

**Figure 2:**
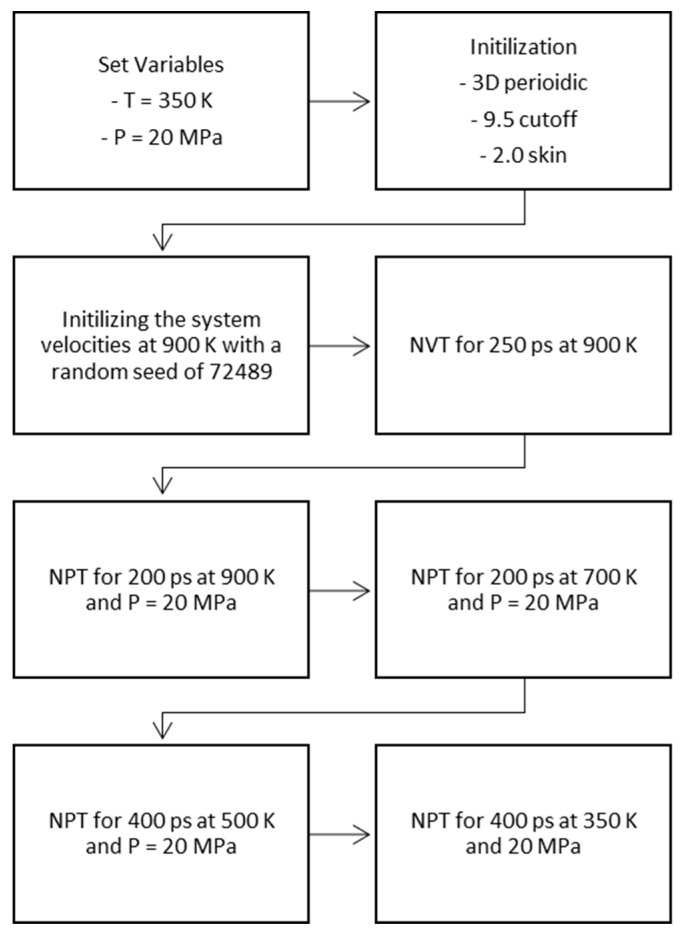
Formation of nanoporous kerogen structures.

**Figure 3:**
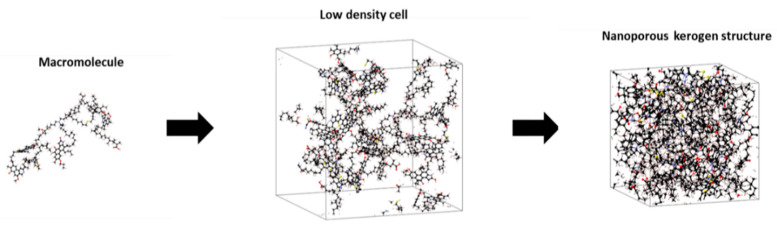
Creation of a nanoporous type II-A kerogen structure from a single macromolecule. The same steps were followed to create the nanoporous structures of the other kerogens.

Kerogens are present in nature as nanoporous structures rather than separated macromolecules. The transformation of the macromolecules in Figure 1 to nanoporous constituents was achieved through molecular modeling. A polymer-compatible forcefield PCFF+ was used to define the atomic force types and charges. Five to seven kerogen macromolecules of each type were placed in a low-density cell (i.e., a density value of ~0.1 g/cm^3^ was used as constrained to create the initial cell). The initial configuration of the system at low density was followed to avoid any instability issues. Temperature and pressure variables were assigned at 350 K and 20 MPa, respectively (i.e., typical reservoir conditions). Then, successive molecular dynamics stages of initialization using three-dimensional periodic boundary, 9.5 cutoff distance and 2.0 skin (isochoric-isothermal NVT and isobaric-isothermal NPT) were performed on the initial cell using LAMMPS, following a systematic temperature reduction to the assigned level (i.e., NVT for 250 ps, two NPT stages for 200 ps followed by two other NPT stages for 400 ps). This annealing approach ensured system stability during convergence [40,41]. The protocol followed in the creation of the nanostructures is given in Figure 2. Four different nanoporous structures corresponding to types II-A, II-B, II-C, and II-D were generated with densities ranging from 1.05 to 1.25 g/cm^3^. An example of the building process is given in Figure 3.

## 5. Adsorption Calculations

**Figure 4:**
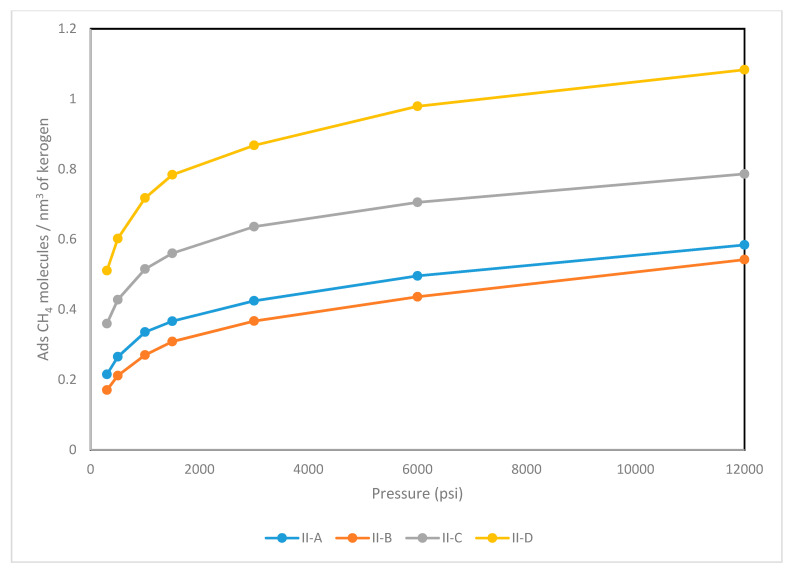
Adsorption profiles of methane for different types of kerogens.

The nanoporous structures developed were examined for their ability to host natural gas (i.e., methane). The temperature was set to at 350 K and a Gibbs ensemble Monte Carlo simulation of the methane-kerogen pair was initiated for pressure stages ranging from 12,000 psi to 300 psi (i.e., 82.68 MPa to 2.067 MPa); these were deemed representative of profiles encountered in the field. At each stage, methane fugacity was determined as a function of pressure and temperature by means of the Peng-Robinson equation of state. The Gibbs ensemble Monte Carlo calculations allowed for tracking of the number of gas molecules trapped in the kerogen bodies. Fugacity data were reported as given in Table 2. The adsorption profiles were then plotted as a function of pressure for kerogens II-A, II-B, II-C, and II-D (see Figure 4). The adsorption calculations were performed using Gibbs Monte Carlo module of MedeA Environment [42].

Generally, the adsorption capacity is controlled by a number of factors including the pressure, temperature, adsorbate and adsorbent (host). The latter, in our case, is the kerogen structure at some predefined maturity levels (i.e., types II-A, II-B, II-C, and II-D). Two properties of kerogen play a major role in determining the adsorption capacity. The first one is the ability of kerogen macromolecules to form a well-defined porous structure. The second factor would be the inter-chemical interactions between adsorbate (methane) and heteroatoms of kerogen structure. As detailed in Section 3, the degree of aromaticity of types II-A, II-B, II-C, and II-D increases with increasing the maturity level. Aromatic rings are present in the form of sheet-like structures. Upon the condensation process described in Section 4, these sheets have higher tendency to resist folding. Hence, they leave some spaces in between that could be occupied with the adsorbate molecules. The adsorption capacity of methane was found to correlate with the fraction of aromatic carbons. Kerogen II-D has the highest adsorption capacity followed by II-C. However, kerogen II-A exhibited slightly larger capacity than II-B despite its lower degree of aromaticity (i.e., Kerogen II-B has 45% while II-A has 41%). That could be explained by the higher percentage II-B has of non-bridging heteroatoms such as aliphatic sulfur, pyrrolic and pyridinic groups. Additionally, oxygen atoms present in kerogen II-B are more aliphatic compared to II-A, which allows them to move more freely, reducing the ability of kerogen to host more methane molecules.

## 6. Comparison with Experimental Data

Three samples collected from an actual shale basin in Saudi Arabia were used to experimentally investigate the relationship between thermal maturity and methane adsorption capacity described in the previous section. The three samples are almost identical in their minerology except for the total organic content TOC (see Table 3). Samples were analyzed for their hydrogen index (HI), oxygen index (OI), and total organic content TOC to qualitatively determine their levels of maturity.

The organic content, TOC, or kerogen content can be determined as follows in Equation (1):

(1) $$\%\;\mathrm{TOC}=\left(0.083\times \frac{\left(S1+S2\right)}{10}\right)+\frac{S4}{10}$$

where S1 is the amount of generated free hydrocarbon obtained after heating the sample to 340 °C in mg/g; S2 is the amount of generated hydrocarbon obtained due to thermal cracking of the kerogen after heating the sample from 340 °C to 640 °C in mg/g; and S4 is the amount of residual carbon after pyrolysis in mg/g.

The hydrogen index (HI) of the sample can be determined as follows in Equation (2):

(2) $$\mathrm{HI}=\frac{100\times S2}{\%TOC}$$

The oxygen index (OI) of the sample can be determined as follows in Equation (3):

(3) $$\mathrm{OI}=\frac{100\times S3}{\%TOC}$$

where S3 is the amount of CO_2_ generated during the thermal breakdown of kerogen in mg CO_2_/g.

A summary of the analyses performed appears in Table 4. Based on the hydrogen indices, it can be qualitatively determined that sample 3 kerogen had the highest level of maturity, followed by samples one and then two respectively.

Then, thermogravimetric analysis was used to measure the adsorption capacity for the three samples at 1000 psi and 100 °C. The results show a clear correlation between adsorption capacity and thermal maturity (see Table 5).

The trend in adsorption capacity is consistent with what was observed through the molecular approach. The adsorption capacity is proportional to the level of maturity.

Sample 3 has the highest adsorption capacity with a total organic carbon TOC content of 2%; this is compared to sample 1 in which TOC content is 4%, but has medium maturity. The result matches the simulation in which the mature kerogen adsorption capacity could be 2.5 times that of the immature kerogen. Sample 3 has the lowest TOC content but the highest methane adsorption capacity, and sample 2 has the highest TOC content but the lowest adsorption capacity because it is immature. This confirmed the conclusion obtained from the molecular simulation study that maturity is the most important factor.

## 7. Numeric Scale for Maturity Level

Decomposition of the four kerogens resulted in different hydrocarbon mixtures. This was verified when they were plotted in a van Krevelen diagram. Such diagrams help to identify different types at different levels of maturity; however, a numeric scale is needed upon which the different kerogen structures can be placed. Kerogens have some degree of polarity determined by their origin (i.e., type) and maturity level.

**Figure 5:**
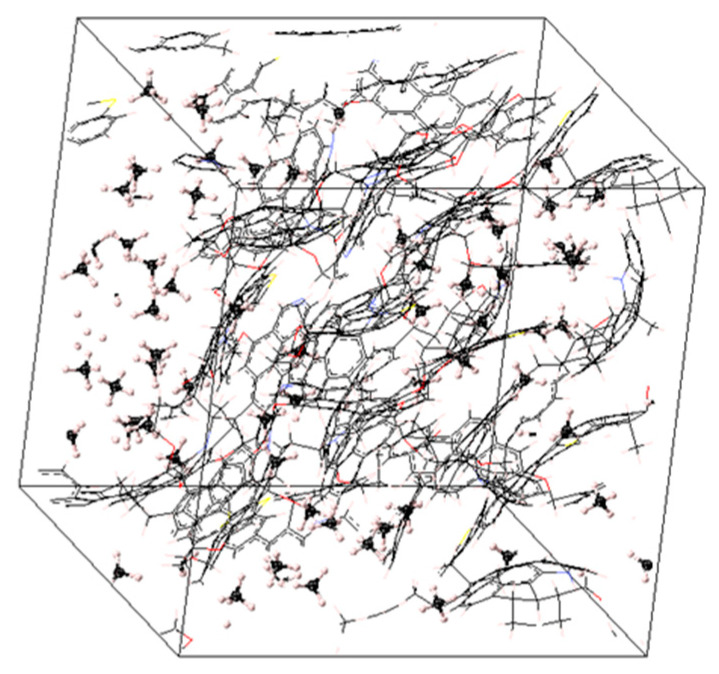
Sample realization of methane molecules adsorbed in II-D kerogen structure.

Kerogen’s degree of polarity is strongly correlated with its thermal maturity. As kerogens undergo thermal cooking, the composition of carbon-hydrogen-oxygen and type of bonding shift to increase the level of polarity. The strategy for obtaining a numeric scale was based on the degree of polarity. In our molecular modeling approach, the four kerogen structures were brought into contact with an equimolar mixture of steam and helium (at 650 K and 20 MPa). While helium is inert, steam is polar. Gibbs Monte-Carlo calculations were performed, and the number of molecules hosted was obtained for each structure (see Figure 5). The fugacities were imposed for water and helium (since they were gas mixtures with relatively high pressures), which was the equivalent of using the chemical potential, as follows in Equation (4):

(4) $$\mu_{i}=\mu_{io}+RTln\left(\frac{f_{i}}{f_{io}}\right)$$

where *μ_i_*, *μ_io_*, *R*, *T*, *f_i_*, and *f_io_* are the chemical potential for component *I*; ideal chemical potential for component *I*; gas constant, temperature, and fugacity of component *I*; and ideal fugacity of component *i*, respectively.

**Figure 6:**
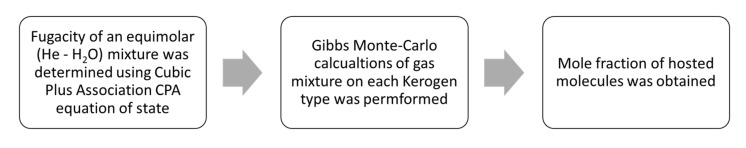
Protocol developed to create the numeric scale of thermal maturity.

The kerogen structures clearly impacted the composition of the molecules hosted. Type II-D, which is the most polar type, had the highest mole fraction of steam, while the other types showed a systematic decrease in the steam mole fraction (see Table 6). This is explained by the increasing affinity of kerogens for steam over helium, the reason for which is most likely the formation of hydrogen bonds between the oxygen atoms of the molecular framework and water molecules of the steam. Types II-A and II-B are from the same type and at very comparable levels of maturity. They share almost the same hydrogen-to-carbon ratios. Both structures were capable of hosting almost the same fraction of helium, with a slightly larger value for type II-A, which is explained by the presence of heteroatoms and their type of bonding. The larger degree of type II-A polarity over type II-B is interestingly matching the adsorption behavior discussed in the previous section. The numeric scale for kerogen maturity level is used in the subsequent section (see Figure 6).

## 8. Modeling of Storage Capacity

**Figure 7:**
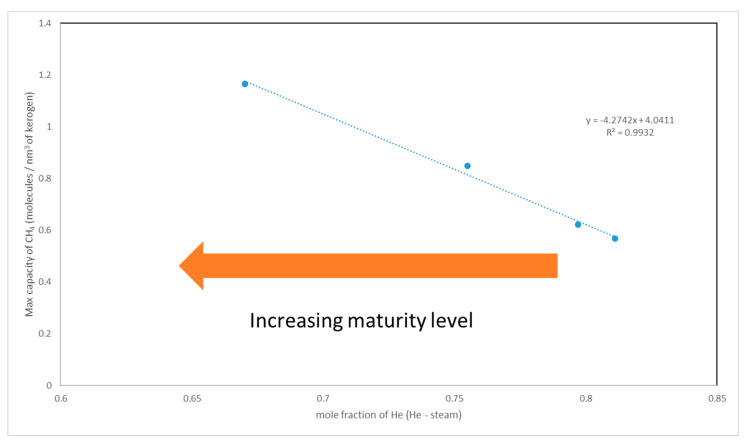
Maximum adsorption capacity and numeric scale of maturity. A linear correlation appears between the He capacity (taken to reflect maturity) and CH_4_ storage capacity.

As the kerogens decomposed, smaller chains of aliphatic hydrocarbons were generated, resulting in the formation of voids (i.e., pores on the nano and angstrom scales). In this study, we observed a correlation between methane adsorption capacity and maturity level (see Figure 4). For instance, kerogen type II-D was found to host around two and half times as much methane as type II-A. The storage capacity appeared to follow a systematic trend with the level of aromaticity, the presence of heteroatoms, and their bonding type. The aforementioned factors give rise to the degree of polarity. The numeric scale of maturity level, which is based on the polarity (described in Section 6), is used to assess the adsorption capacity of kerogens as a function of thermal maturity. Each kerogen type had a unique value along the scale. We plotted those values against the maximum adsorption capacity, as shown in Figure 7.

The empirical mathematical relationship can be given as (Equation (5)):

(5) $$C_{CH4}=-4.27\;Y_{He\;\left(He-H2O\right)}+4.04$$

where *C*_*CH*4_ is the maximum adsorption capacity of the methane. The unit of molecules of methane is per nm^3^ of kerogens. The *Y*_*He*(*He–H2O*)_ variable is the numeric scale of maturity level introduced in Section 6.

## 9. Conclusions

In this article, a molecular modeling study of the relationship between the thermal maturity and storage capacity of kerogens was presented. Four prototypes of kerogen molecules covering a wide range of maturity levels were used to build nanoporous structures mimicking those naturally occurring in source rocks. A numeric scale based on the concept of polarity assessment was created and Gibbs Monte Carlo adsorption calculations performed, considering typical pressure profiles encountered during reservoir depletion. It was found that the adsorption capacity correlated with the degree of maturity. A mathematical relationship between the maximum adsorption capacity and thermal maturity was obtained that could help with reserve estimations. The correlation observed between the thermal maturity and adsorption capacity of kerogens was found to be consistent with experimental adsorption calculations performed on shale samples collected from the actual shale gas basin. Experimental and simulation results showed that methane gas adsorption capacity is strongly correlated to the kerogen maturity not the kerogen content.

## Figures and Tables

**Table 1:** Volumetric and Material Balance Equations MBE Models for Nanopores of Source Rocks.

Model	Equation
Ambrose et al. [2]	$G_{st}=G_{f}+G_{a}$
	$G_{f}=\frac{32.0368}{B_{g}}\left[\frac{\phi (1-S_{w})}{\rho_{b}}-\frac{1.318\times 10^{-6}\hat{M}}{\rho_{s}}\left(G_{sL}\frac{p}{p+p_{L}}\right)\right]$
	$G_{a}=G_{sL}\frac{p}{p+p_{L}}$
King [10]	$G_{p}=\frac{V_{b2}\phi_{i}z_{sc}T_{sc}}{p_{sc}T}\left(\frac{p_{i}}{z_{i}^{*}}-\frac{p}{z^{*}}\right)$
	$z^{*}=\frac{z}{\left(\left[1-c_{\phi }\left(p_{i}-p\right)\right]\left(1-{\bar{S}}_{w}\right)+\frac{zRTC_{ME}}{\phi_{i}p}\right)}$
Jensen & Smith [11]	$\frac{p}{p_{L}+p}=\frac{-1}{1.3597V_{L}Ah\rho_{c}}G_{P}+\frac{p}{p_{L}+p_{i}}$
Clarkson & McGovern [12]	$\frac{p}{p_{L}+p}+\frac{32,037\phi (1-{\bar{S}}_{w})}{V_{L}B_{g}\rho_{c}}=\frac{-0.7355}{V_{L}Ah\rho_{c}}G_{P}+\left[\frac{p_{i}}{p_{L}+p_{i}}+\frac{32,037\phi (1-S_{wi})}{V_{L}B_{gi}\rho_{c}}\right]$
Kinniburgh [13]	$q=\frac{Q_{m}k_{b}p}{\left(p_{s}-p\right)\left[1+\left(k_{b}-1\right)\frac{p}{p_{s}}\right]}$
Mahmoud [14]	$G=V_{B}\left[\left(\frac{\varphi S_{g}}{B_{gi}}\right)+\frac{Q_{m}k_{b}p_{i}}{\left(p_{s}-p_{i}\right)\left[1+\left(k_{b}-1\right)\frac{p_{i}}{p_{s}}\right]}\left(1-\varphi \right)\frac{\rho_{ma}}{\rho_{gas}}\right]$
Mengal [15]	$G=V_{B}\left[\left(\frac{\varphi S_{g}}{B_{gi}}\right)+\left(V_{L}\frac{p_{i}}{p_{i}+p_{L}}\right)\right]$ based on the Langmuir isotherm
	$G=V_{B}\left[\left(\frac{\varphi S_{g}}{B_{gi}}\right)+\left(k_{f}p_{i}^{n}\right)\left(1-\varphi \right)\frac{\rho_{ma}}{\rho_{gas}}\right]$ based on the Freundlich isotherm
	$G=V_{B}\left[\left(\frac{\varphi S_{g}}{B_{gi}}\right)+\frac{Q_{m}k_{b}p_{i}}{\left(p_{s}-p_{i}\right)\left[1+\left(k_{b}-1\right)\frac{p_{i}}{p_{s}}\right]}\left(1-\varphi \right)\frac{\rho_{ma}}{\rho_{gas}}\right]$ based on the BET adsorption isotherm

**Table 2:** Fugacity data used in the adsorption calculations.

P (MPa)	Fugacity (MPa)
2.067	2.028
3.445	3.340
6.890	6.494
10.335	9.503
20.670	18.056
41.340	35.842
82.680	86.428

**Table 3:** Minerology of the three shale samples.

Mineral (%)	Shale 1	Shale 2	Shale 3
Quartz	31	31	32
Clay minerals	44	42	43
Feldspars	11	11	12
Pyrite	7	8	9
Carbonate	3	2	2
TOC	4	6	2

**Table 4:** Maturity Assessment of the actual downhole Samples.

Property	Sample 1	Sample 2	Sample 3
S1, mg/g	0.12	1.04	0.16
S2, mg/g	4.5	10.1	0.65
S4, mg/g	2	4.3	0.76
T_max_, °C	430	420	490
TOC, %	4	6	2
OI	55	64	35
HI	120	150	30

**Table 5:** Adsorption Capacity of the Actual Downhole Samples.

Kerogen Maturity	Max Methane Adsorption at 1000 psi and 100 °C (mg CH4/g Shale Rock)
Mature Kerogen (Sample 3)	200
Medium Maturity (Sample 1)	100
Immature Kerogen (Sample 2)	40

**Table 6:** Numeric Scale of Thermal Maturity.

Kerogen Type	H/C	O/C	Mole Fraction of He
II-A	1.1600	0.0950	0.7970
II-B	1.1200	0.0590	0.8111
II-C	0.9050	0.0537	0.7549
II-D	0.5820	0.0514	0.6701

## Nomenclature

*G_f_*	Free gas (SCF/ton)
*G_a_*	Adsorbed gas (SCF/ton)
*G_SL_*	Maximum adsorbed gas (SCF/ton)
*G_m_*	Maximum adsorbed gas (SCF/ton)
*G_p_*	Produced gas (SCF)
*S_w_*	Water saturation
*S_g_*	Gas saturation
*μ*	Gas viscosity (cP)
*M*	Molecular weight (g/mol)
*R*	Gas constant (8.314 J/mol·K)
*T*	Temperature (R or K)
*P*	Pressure (psi or Pa)
*P_L_*	Langmuir pressure (psi or Pa)
*P_S_*	Saturation pressure (psi or Pa)
*k_b_*	BET adsorption constant
*k_f_*	Freundlich adsorption constant
*ρ_c_*	Density of coalbed methane (g/cm^3^)
*Z^*^*	Modified compressibility factor
*C_ME_*	Molar adsorption capacity (lbm.mol/ft^3^)
*C_g_*	Gas compressibility coefficient (1/psi)
*V_SL_*	Maximum sorption capacity (ft^3^ or cm^3^)
*ρ_s_*	Sorbed phase density (g/cm^3^)
*ρ_b_*	Bulk density (g/cm^3^)
*ρ_ma_*	Matrix density (g/cm^3^)
*ρ_gas_*	Gas density (g/cm^3^)
*B_g_*	Formation volume factor (ft^3^/SCF)
*V_b_*	Bulk volume (ft^3^)

## References

[1] Kang S.M., Fathi E., Ambrose R.J., Akkutlu I.Y., Sigal R.F. (2011). Carbon Dioxide Storage Capacity of Organic-Rich Shales. SPE J..

[2] Ambrose R.J., Hartman R.C., Campos M.D., Akkutlu I.Y., Sondergeld C.H. (2012). Shale Gas-in-Place Calculations Part I: New Pore-Scale Considerations. SPE J..

[3] Santos J.M., Akkutlu I.Y. (2013). Laboratory Measurement of Sorption Isotherm under Confining Stress with Pore-Volume Effects. SPE J..

[4] Hou Y., Wang J., Harris N.B., Pedersen P., He S., Cheng C., Li Y. (2015). Preliminary study on the pore characterization of lacustrine shale reservoirs using low pressure nitrogen adsorption and field emission scanning electron microscopy methods: A case study of the Upper Jurassic Emuerhe Formation, Mohe basin, northeastern China. Can. J. Earth Sci..

[5] Zhou S., Yan G., Xue H., Guo W., Li X. (2016). 2D and 3D nanopore characterization of gas shale in Longmaxi formation based on FIB-SEM. Mar. Pet. Geol..

[6] Klaver J., Desbois G., Littke R., Urai J.L. (2016). BIB-SEM pore characterization of mature and post mature Posidonia Shale samples from the Hils area, Germany. Int. J. Coal Geol..

[7] Chen S., Han Y., Fu C., Zhang H., Zhu Y., Zuo Z., Shangbin C., Yufu H., Changqin F., Han Z. (2016). Micro and nano-size pores of clay minerals in shale reservoirs: Implication for the accumulation of shale gas. Sediment. Geol..

[8] Jiao K., Yao S., Liu C., Gao Y., Wu H., Li M., Tang Z. (2014). The characterization and quantitative analysis of nanopores in unconventional gas reservoirs utilizing FESEM–FIB and image processing: An example from the lower Silurian Longmaxi Shale, upper Yangtze region, China. Int. J. Coal Geol..

[9] Yang C., Zhang J., Han S., Xue B., Zhao Q. (2016). Classification and the developmental regularity of organic-associated pores (OAP) through a comparative study of marine, transitional, and terrestrial shales in China. J. Nat. Gas Sci. Eng..

[10] King G. (1993). Material-Balance Techniques for Coal-Seam and Devonian Shale Gas Reservoirs with Limited Water Influx. SPE Reserv. Eng..

[11] Jensen D., Smith L.K. A Practical Approach to Coalbed Methane Reserve Prediction using a Modified Material Balance Technique. Proceedings of the International Coalbed Methane Symposium.

[12] Clarkson C.R., Bustin M. (2011). Coalbed Methane: Current Field-Based Evaluation Methods. SPE Reserv. Eval. Eng..

[13] Kinniburgh D.G. (1986). General purpose adsorption isotherms. Environ. Sci. Technol..

[14] Mahmoud M. (2019). Effect of Gas Adsorption on the Estimation of Gas in Place (GIP) in Conventional and Unconventional Reservoirs. Arab. J. Sci. Eng..

[15] Mengal S.A. (2010). Accounting for adsorbed gas and its effect on production behavior of shale gas reservoirs. Master’s Thesis.

[16] Seidle J.P. A Modified p/Z Method for Coal Wells. Proceedings of the SPE Rocky Mountain Regional Meeting.

[17] Zhang M., Zhang Y., Yang L., Mei H., Shen P., Ge J. (2017). A New Method to Calculate the Recoverable Reserves and Recovery Ratio of Shale Gas Reservoir. Open J. Yangtze Oil Gas.

[18] Hu S., Hu X., He L., Chen W. (2019). A New Material Balance Equation for Dual-Porosity Media Shale Gas Reservoir. Energy Procedia.

[19] Liu K., Ostadhassan M., Zhou J., Gentzis T., Rezaee R. (2017). Nanoscale pore structure characterization of the Bakken shale in the USA. Fuel.

[20] Tang X., Jiang Z., Jiang S., Wang P., Xiang C. (2016). Effect of Organic Matter and Maturity on Pore Size Distribution and Gas Storage Capacity in High-Mature to Post-Mature Shales. Energy Fuels.

[21] Zhang Y., He Z., Jiang S., Lu S., Xiao D., Chen G., Zhao J. (2018). Factors Affecting Shale Gas Accumulation in Overmature Shales Case Study from Lower Cambrian Shale in Western Sichuan Basin, South China. Energy Fuels.

[22] Guo X., Huang Z., Ding X., Chen J., Chen X., Wang R. (2018). Characterization of Continental Coal-Bearing Shale and Shale Gas Potential in Taibei Sag of the Turpan-Hami Basin, NW China. Energy Fuels.

[23] Down A.L., Himus G.W. (1941). A Preliminary Study of the Chemical Constitu tion of Kerogen. J. Inst. Pet..

[24] Forsman J., Hunt J. (1958). Insoluble organic matter (kerogen) in sedimentary rocks. Geochim. Cosmochim. Acta.

[25] Van Krevelen D.W. (1961). Coal: Typology, Chemistry, Physics, Constitution.

[26] Durand B., Espitalie J. (1973). Evolution de la matie’re organique au cours de l’enfouissement des se’diments. C. R. L’Acad. Sci..

[27] Tissot B.D.B. (1974). Influence of Nature and Diagenesis of Organic Matter in Formation of Petroleum. AAPG Bull..

[28] Durand B., Nicaise G., Roucache´ J., Vandenbroucke M., Hagemann H.W. (1975). Geochemical study of a series of coals. Advances in Organic Geochemistry.

[29] Tissot B., DeRoo G., Hood A. (1978). Geochemical study of the Uinta Basin: Formation of petroleum from the Green River formation. Geochim. Cosmochim. Acta.

[30] Tissot B.P., Vandenbroucke M. (1983). Geochemistry and Pyrolysis of Oil Shales. Proceedings of the Pedagogic Roles of Animations and Simulations in Chemistry Courses.

[31] Albrecht P., Vandenbroucke M., Mandengué M. (1976). Geochemical studies on the organic matter from the Douala Basin (Cameroon)—I. Evolution of the extractable organic matter and the formation of petroleum. Geochim. Cosmochim. Acta.

[32] Forsman J.P., Breger I.A. (1963). Geochemistry of kerogen. Organic Geochemistry, Earth Series Monograph.

[33] Burlingame A.L., Haug P.A., Schnoes H.K., Simoneit B.R., Schenck P.A., Havenaar I. (1969). Fatty acids derived from the Green River Formation oil shale by extractions and oxidations—A review. Advances in Organic Geochemistry 1968, Proceedings of the 4th International Meeting on Organic Geochemistry, Amsterdam, Netherlands, 16–18 September 1968.

[34] Djuricic M., Murphy R., Vitorovic D., Biemann K. (1971). Organic acids obtained by alkaline permanganate oxidation of kerogen from the Green River (Colorado) shale. Geochim. Cosmochim. Acta.

[35] Yen T. (1976). Chapter 7 Structural Aspects of Organic Components in Oil Shales. Pressure Transient Formation and Well Testing: Convolution, Deconvolution and Nonlinear Estimation.

[36] Vandenbroucke M., Largeau C. (2007). Kerogen origin, evolution and structure. Org. Geochem..

[37] Vandenbroucke M. (1980). Kerogen, Insoluble Organic Matter from Sedimentary Rocks.

[38] Behar F., Vandenbroucke M. (1987). Chemical modelling of kerogens. Org. Geochem..

[39] Ungerer P., Collell J., Yiannourakou M. (2014). Molecular Modeling of the Volumetric and Thermodynamic Properties of Kerogen: Influence of Organic Type and Maturity. Energy Fuels.

[40] AlAfnan S., Sultan A.S., Aljaberi J. (2020). Molecular Fractionation in the Organic Materials of Source Rocks. ACS Omega.

[41] Al-Afnan S., Al Jawad M., Glatz G., Sultan A.S., Windiks R. (2020). Sustainable Production from Shale Gas Resources through Heat-Assisted Depletion. Sustainability.

[42] Yiannourakou M., Ungerer P., Leblanc B., Ferrando N., Teuler J.-M. (2013). Overview of MedeA®-GIBBS capabilities for thermodynamic property calculation and VLE behaviour description of pure compounds and mixtures: Application to polar compounds generated from ligno-cellulosic biomass. Mol. Simul..

